# Gemcitabine combined with oxaliplatin in pretreated patients with malignant pleural mesothelioma: an observational study

**DOI:** 10.1186/1745-6673-3-34

**Published:** 2008-12-18

**Authors:** Athanasios Xanthopoulos, Torsten T Bauer, Torsten G Blum, Jens Kollmeier, Nicolas Schönfeld, Monika Serke

**Affiliations:** 1 Respiratory Diseases Clinic Heckeshorn, Department of Pneumology, HELIOS Klinikum Emil von Behring, Berlin, Germany

## Abstract

**Background:**

The aim of this study was to investigate the efficacy and safety of oxaliplatin ± gemcitabine in patients with diffuse malignant pleural mesothelioma (MPM) pretreated with pemetrexed.

**Methods:**

The study enrolled consecutive patients with relapsed MPM, all of them pretreated with a platin-pemetrexed-based chemotherapy. Oxaliplatin 80 mg/m^2 ^was administered as monotherapy or in combination with gemcitabine 1000 mg/m^2 ^given on day 1 and 8. Cycles were repeated every 21 days. The primary endpoints were response rate and disease control rate. Secondary endpoints included overall survival (OS), time to tumour progression (TTP), progression-free survival (PFS), time to treatment failure (TTF), and toxicity.

**Results:**

Between February 2005 and September 2007 29 patients (median age: 65.0 years, World Health Organisation (WHO) performance status: 0–3) were enrolled. The follow-up period encompassed 5.4 to 97.4 weeks (median: 24.3 weeks). Out of these 29 patients, 15 were treated in second, 10 in third, 3 in fourth and 1 in fifth line, respectively. The majority of the patients received the combination oxaliplatin and gemcitabine (n = 25 vs. 4; 86.2 vs. 13.8%).

The median overall survival (OS) was 71.7 weeks (30.6–243.3 weeks), whereas survival from the start of oxaliplatin/gemcitabine-treatment was 24.3 weeks (5.4–97.3 weeks). Median time to tumour progression (TTP) was 9.3 weeks (3.0–67.6 weeks).

Partial response (PR) was observed in 2 patients (6.9%), stable disease (SD) for at least three courses of treatment in 11 patients (37.9%). Thus, disease control rate was 44.8%, whereas 16 of 29 patients exhibited progressive disease (55.2%).

The toxicity profile was favourable, with no WHO grade 4-toxicities, only few dose-reductions were performed due to non-symptomatic haematotoxicities (neutropenia, thrombopenia). Mild WHO grade 2 neurotoxicity was seen in 6 patients.

**Conclusion:**

Pemetrexed-pretreated patients with progressive MPM may benefit from a consecutive chemotherapy with oxaliplatin and gemcitabine without significant toxicity.

## Background

Malignant mesothelioma (MM) is a rare malignant disease and originates from neoplastic mesothelial cells, hence, primarily situated to serous membranes of pleura, peritoneum, pericardium, or testis. Though exhibiting an aggressive, locally advancing growth pattern, MM expands clinically unperceived for a long time [1-3]. The age-adjusted incidence of mesothelioma in 11 industrialized countries has been recently estimated with 14 to 35 cases per million per year [4]. In autopsy studies, the frequency of malignant mesothelioma varies from 0.02 to 0.7%, with a rate of 0.2% in the largest series. The pleura is more often involved than the peritoneum, with a predominance of the right over the left pleura (60:40) [5]. Most cases of MM can be ascribed to chronic occupational exposure against asbestos fibres. [6]. Overall, the prognosis for patients with malignant pleural mesothelioma (MPM) is poor. Accordingly, several studies reported consistently 5-year survival rates of less than 1% and revealed low median survival times for therapy-naive patients of approximately 8 to 12 months [3,7-9].

Formerly, mesothelioma was thought to be resistant to chemotherapy. With the initiation of pemetrexed in 2002, chemotherapy with platin/pemetrexed chemotherapy became standard for MPM treatment with response rates of approximately 40%. However, patients relapse during or after a platin/pemetrexed-based chemotherapy and therefore may benefit from a complementary chemotherapy [10].

Gemcitabine (2,2'-difluorodesoxycytidine) is a pyrimidine analogue with activity against a wide range of solid tumours, including pancreatic carcinoma and non small cell lung carcinoma [11]. Its mechanism of action, toxicity, and clinical pharmacology have been reviewed extensively [12,13]. Moreover, gemcitabine seems to be active in MPM [10]. The enantiomer oxaliplatin (Cis-[oxalate]trans-l-1,2-Diaminocyclohexan) is an antineoplastic substance belonging to the class of platin-derivats. Oxaliplatin has demonstrated activity in the treatment of advanced tumours, both in combined and in monotherapeutic regimes. Notably, oxaliplatin has proven to be active at least in vitro against cisplatin-resistant cell lines and exhibits clinical activity in the treatment of cisplatin/carboplatin refractory diseases [14-16].

Schütte and co-workers have previously introduced a combined chemotherapy with oxaliplatin and gemcitabine as an active first line therapy in MPM [17]. The primary aim of this study was to evaluate the efficacy as well as safety of oxaliplatin in combination with gemcitabine or as a monotherapy in patients with MPM who were preteated with a pemetrexed/platin-combination therapy.

## Methods

### Inclusion criteria

All consecutive patients with histologically confirmed advanced MPM and progressive disease after pemetrexed/platin-based chemotherapy were enrolled in this study. Further inclusion criteria were: age < 80 years and life expectancy > 2 months, unidimensional measurable disease, World Health Organisation (WHO) performance status of 0–2, and adequate haematologic (absolute neutrophil ≥ 1.5 × 10^9^/L, platelets ≥ 100 × 10^9^/l, and hemoglobin ≥ 9 g/dL), hepatic (bilirubin ≤ 1.2 mg/dL, transaminases and cholestatic parameters within 2-fold of upper limit of reference range), and renal (creatinine clearance ≤ 45 mL/min according to Cockcroft and Gault) functions. Previous surgery or radiotherapy was allowed in this study.

### Pretreatment evaluation

All patients underwent an accurate medical history. In addition to a detailed history of working activities and asbestos exposure, patients underwent a physical examination, a complete blood count and biochemistry profile, an ECG, chest X-Ray and chest and upper abdomen CT scan. Physical examinations were also performed before each treatment cycle. Bone scan, brain magnet resonance imaging and upper abdominal ultrasound were performed only if indicated clinically.

### Study medication

Initially, all patients received oxaliplatin at a dose of 80 mg/m^2 ^with or without gemcitabine at a dose of 1000 mg/m^2 ^according to the scheme proposed by Schütte and co-workers [17]. Gemcitabine was diluted in normal saline and administered intravenously over 30 minutes on days 1 and 8 of each 21-day cycle. Oxaliplatin at a dose of 80 mg/m^2 ^was diluted in 5% glucose and administered intravenously in 3 h on days 1 and 8 of each 21-cycle after the gemcitabine-administration. Antiemetic prophylaxis was performed with 5-hydroxytryptamine (HT3) antagonists. Additional antiemetic agents were used according to the advice of the treating physician.

The response evaluation was monitored by Chest-X-Ray after each cycle and computed tomography scan (CT) after 2 or 3 cycles. Treatment in responding patients or patients with stable disease was continued for a maximum of six cycles. Both agents were reduced on day 8 to 25% in the event of grade 3 leukopenia and/or the thrombopenia between 99 × 10^9^/L and 50 × 10^9^/L. Therapy was omitted if the absolute neutrophil count (ANC) was less than 0.5 × 10^9^/L or if grade 3 or 4 non-haematologic toxicity occurred. Treatment was repeated on day 21 if the ANC was greater than or equal to 1.5 × 10^9^/L and the platelet count was greater than or equal to 100 × 10^9^/L. Treatment was delayed for one week if the ANC and/or platelet counts fell below these levels.

### Response and toxicity evaluation

Tumour response was assessed according to RECIST criteria with evaluation of target lesions at baseline, after every cycle, and at the end of treatment [18]. Nodular thickening of the pleura was accepted as a target lesion if the thickening was at least 2 cm in its greatest perpendicular diameter. CT-scans were mandatory for evaluation of intrathoracic lesions.

The primary efficacy endpoints were response rate (differentiated in complete response (CR), partial response (PR), stable disease (SD), and progressive disease (PD)) and disease control rate (CR+PR+SD), secondary endpoints were: overall survival (OS), which was defined as time from first diagnosis of MPM to death, survival after start of treatment (defined as time from first application of oxaliplatin with or without gemcitabine to death), time to tumour progression (TTP; defined as time from first application of oxaliplatin with/without gemcitabine to first observation of PD), progression-free survival (PFS; defined as time from first application of oxaliplatin with or without gemcitabine to first observation of PD or death), time to treatment failure (TTF; defined as time from first application of oxaliplatin with or without gemcitabine to first observation of PD, death, or discontinuation of therapy), and toxicity.

### Statistics

All frequencies are reported as number and percentage. Continuous variables are reported as mean ± standard deviation and due to the observational design of this study also as median ± range. Survival analyses were compute as Kaplan-Meier plots with the Statistical package for Social Sciences (SPSS^®^) on a Microsoft Windows^® ^operating system.

## Results

Between February 2005 and September 2007 overall 29 patients (mean age 64.6 years, median 65.0 years, World Health Organization (WHO) performance status 0–2) were enrolled. Detailed patients' characteristics are listed in table 1.

**Table 1:** Patients' characteristics

**Patient Characteristics (n = 29)**	**No. (%)**
**Gender**	

Male	27 (93.1%)

**WHO performance status**	

0	5 (17.2%)

1	18 (62.1%)

2	3 (10.3%)

3	3 (10.3%)

**Asbestos exposure**	

Yes	17 (58.6%)

No	1 (3.4%)

Not defined	11 (37.9%)

**Histologic Type**	

Epithelial	27 (93.1%)

Sarcomatoid	1 (3.4%)

Biphasic	1 (3.4%)

The median interval from the primary diagnosis to the beginning of the study treatment was 53.4 weeks (7.9–185.4 weeks). The follow-up period varied from 5.4 to 97.4 weeks (mean 30.0 weeks, median 24.3 weeks). All patients were pretreated with a platin/pemetrexed-combination. Out of these 29 patients, 15 were then treated (within this study) in second (51.7%), 10 in third (34.5%), 3 in fourth (10.3%), and 1 in fifth line (3.4%), respectively. Every patient received at least one cycle of chemotherapy of oxaliplatin with/without gemcitabine. In the majority of cases oxaliplatin and gemcitabine were administered as combination therapy (n = 25 patients vs. 4 patients with oxaliplatin monotherapy; 86.2 vs. 13.8%). The number of chemotherapy cycles ranged from 1 to 6 (mean: 3.0 cycles; median: 3 cycles).

**Figure 1:**
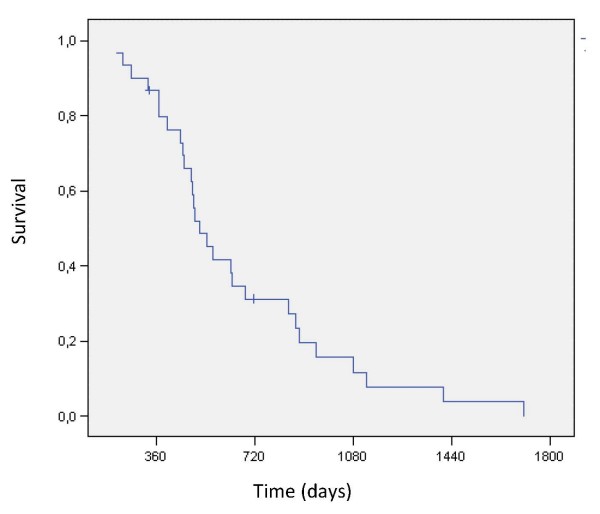
**Kaplan-Meier-plot for overall survival**.

**Figure 2:**
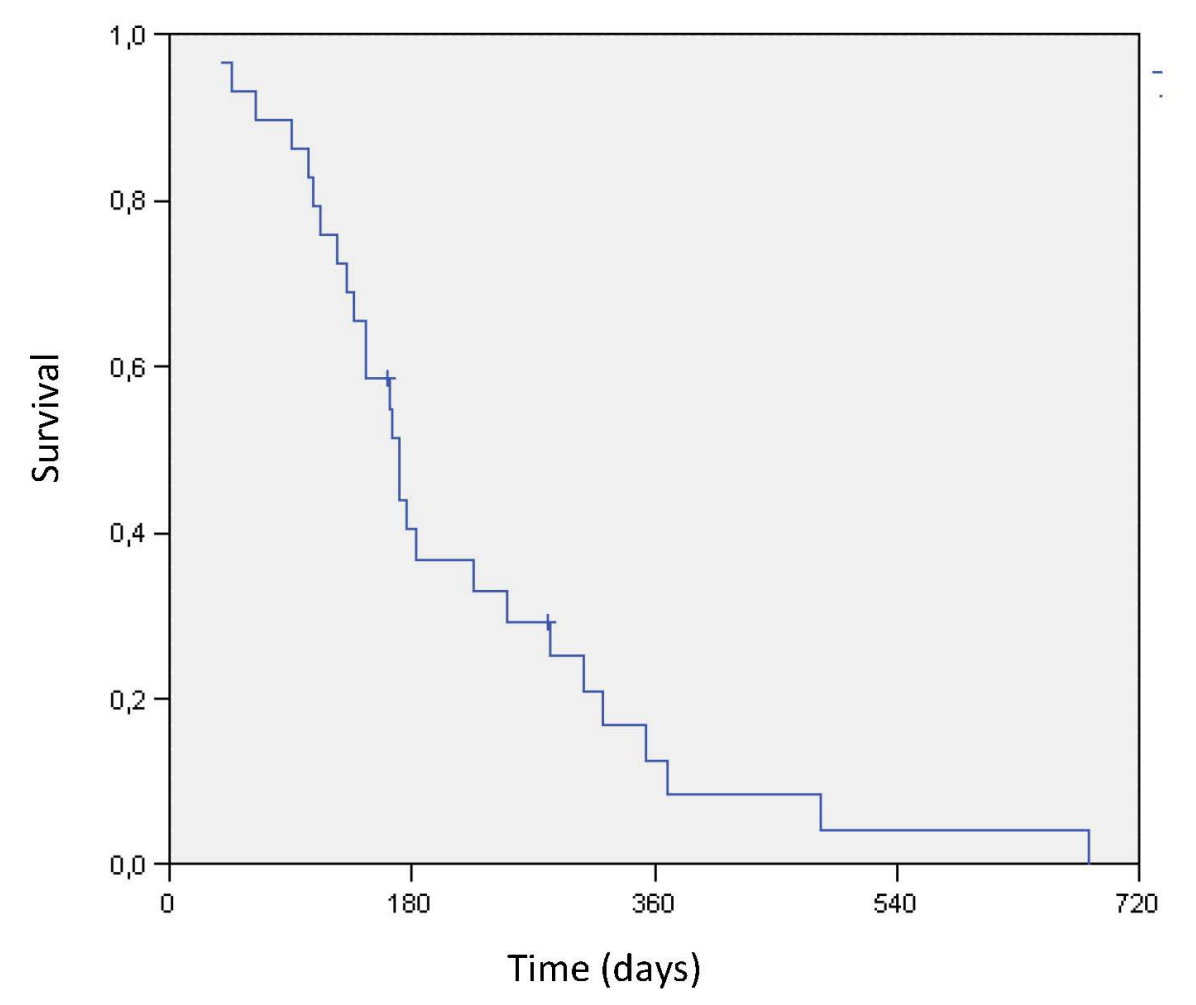
**Kaplan-Meier-curve for survival from the start of Oxaliplatin with or without gemcitabine**.

All patients were assessable for response evaluation. The median OS (from diagnosis of MPM) was 71.7 weeks (30.6–243.3 weeks), whereas survival from the start of treatment was 24.3 weeks (5.4–97.3 weeks). Median TTP added up to 9.3 weeks (3.0–67.6 weeks). PFS and TTF were equally calculated as 11.7 weeks (3.0–50.4 weeks), since no therapy had to be discontinued for other reasons than PD. Kaplan-Meier plots for OS and survival from the start of treatment are displayed in figure 1 and 2, respectively.

Partial remission (PR) was observed only in 2 patients (6.9%), stable disease (SD) for at least three courses of treatment in 11 patients (37.9%). Thus, disease control rate was 44.8%, whereas 16 of 29 patients suffered primarily progressive disease (55.2%).

A more differentiated depiction of response to current chemotherapy is given in table 2, correlating it with numbers of previous chemotherapy regimens, response to previous chemotherapy, and current chemotherapy regimen (combination or monotherapy).

**Table 2:** Response to current chemotherapy with regard to previous chemotherapy regimens, response to previous chemotherapy, and current chemotherapy regimen (combination or monotherapy).

	Numbers of previous chemotherapy regimens				Response to previous chemotherapy			Current chemotherapy regimen	
Response to current chemotherapy	1	2	3	4	PR	SD	PD	Oxaliplatin and gemcitabine	Oxaliplatin

Partial response	2	0	0	0	0	1	1	2	0

Stable disease	6	5	0	0	0	3	8	10	1

Progressive disease	7	5	3	1	0	3	13	13	3

The toxicity profile was included no WHO grade 4-toxicities and only few dose-reductions were performed due to non-symptomatic haematotoxicity (neutropenia, thrombopenia): altogether, 14 episodes of WHO grade 2–3 neutropenia were reported in 10 and 10 episodes of WHO grade 2–3 thrombocytopenia in 10 patients, respectively. All haematologic adverse events were rapidly reversible and did not result in life-threatening complications (e.g. sepsis or hemorrhage). Minor anemia (WHO grade 1–2) developed in 3 patients. Mild WHO grade 2-neurotoxicity was seen in 6 patients (20.7%), renal toxicity was evident only in 1 patient (3.4%). Besides, 4 patients complained of fatigue (13.7%; WHO grade 1–2).

## Discussion

The major results of our study were: (1) Partial remission (PR) was unlikely (6.9%) when oxaliplatin/gemcitabine was used as chemotherapy in multiple pretreated, pemetrexed-resistent patients with MPM. (2) However, disease control could be achieved in 44.8%, when at least three cycles of chemotherapy could be applicated. (3) The combination therapy was well tolerated and no WHO grade 4-toxicities occurred in our patients.

The diagnosis of malignant pleural mesothelioma (MPM) is still associated with a poor prognosis. The 5-year survival rates are less than 1% and median survival has been reported to be as low as 8 to 12 months for therapy-naïve patients [3,7-9]. The introduction of pemetrexed/platin-based chemotherapy has improved therapeutic options in the treatment of MPM. However, no chemotherapy regime could so far demonstrate a significant efficacy in patients with relapsed MPM after pemetrexed/platin-based treatment [10].

Vogelzang and co-workers showed an evident benefit of a pemetrexed/cisplatin-based chemotherapy for the treatment of MPM with a response rate of 41.3% in contrast to 16.7% response in the control group with cisplatin-monotherapy [19]. A decade ago, a review by this group figured response rates of MPM to different chemotherapy-regimes regularly as low as 10 to 20%, with only single, unconfirmed studies indicating possible higher response rates [1]. The more recent review by Ellis and associates concluded, that among other chemotherapeutics for MPM, older studies with gemcitabine monotherapy revealed response rates from 0 up to 31% [10]. Schütte and his colleagues were the first to describe results of the oxaliplatin/gemcitabine combination for the first line chemotherapy of MPM. In their multicenter phase II study, objective responses were measured as follows: partial response in 40% (10/25 patients), stable disease in 24% (6/25 patients), and progressive disease in 36% (9/25 patients). Median TTP and OS were 7 and 13 months, respectively.

Since pemetrexed based therapy has become first line treatment for MPM, we report in our observational study the efficacy and safety of the previously described oxaliplatin/gemcitabine protocol for further therapy in pemetrexed/platin pretreated patients. Our rationale for selecting these two agents was first, the favourable response rate in first line therapy for MPM demonstrated by Schütte and co-workers [17], and second, the acceptable toxicity-profile and thus possible clinical benefits [11-17].

All 29 patients included in our study had advanced MPM with a median interval from primary diagnosis to begin of study treatment of 53 weeks (8 – 185 weeks) and 1 to 4 preceding chemotherapies (including the pemetrexed/platin combination). The median OS (beginning from primary diagnosis) was 72 weeks (31 – 243 weeks) and the median survival after beginning of study treatment 24 weeks (5 – 97 weeks), respectively. The median TTP was 9 weeks (3 – 68 weeks). PFS and TTF were both 12 weeks (3 – 50 weeks). Partial remission (PR) was expectably low (6.9%), but overall disease control for at least three cycles of chemotherapy could be achieved in 44.8%.

Generally, evidence is lacking for second line chemotherapy in relapsing MPM in literature. A most recently published paper by Jassem and his group compared the outcome of pemetrexed in second line treatment with best supportive care (BSC) in previously treated patients. In this trial median survival after enrolment was 14.5 months in the pemetrexed plus BSC group vs. 9.7 months in the BSC group (p = 0.74). TTP, PFS and TTF differed significantly in both groups, favouring the pemetrexed group [20].

In a post-study analysis, Vogelzang and co-workers determined the benefit of a second-line chemotherapy in a subgroup of patients treated with pemetrexed/cisplatin or cisplatin. They found a prolonged survival following a second line chemotherapy. However, whether this effect could be attributed to the second line chemotherapy or the prolonged survival caused by the natural history of the tumour could not be demonstrated with statistical significance. Agents used for second line chemotherapy included gemcitabine, vinorelbine, doxorubicin, and epirubicin in mono- or combination therapy [21].

Porta and colleagues investigated the anti-tumour activity of raltitrexed and oxaliplatin given as a second line regime in a series of 14 patients. The study was prematurely discontinued because no response according to the given endpoints response rate, TTP and OS was observed [22]. In contrast, Fizazi and his group observed a median survival of 44 weeks from the start of treatment and a median survival of 226 weeks from the primary diagnosis in 15 patients receiving a second line chemotherapy with the same regime of raltitrexed and oxaliplatin [23].

The toxicities of the combination oxaliplatin/gemcitabine as well as of the monotherapy with oxaliplatin were very acceptable in our study – in accordance with the low toxicities reported in the first line therapy [17]. In general, chemotherapy was tolerated well by all patients. Mild haematologic toxicities were common but not associated with complications or even life-threatening events. Solely peripheral neuropathy, fatigue and mild renal impairment were observed.

This study has limitations due to its observational design and heterogeneity of the patients included. However, no standard therapy has been established as second line or beyond and a structured observational approach may help to foster the understanding of efficacy and safety in this clinical setting. Second, we reported all patients receiving oxaliplatin/gemcitabine therapy at any stage of the disease. This method may reduce applicability of your results in terms of efficacy as a second line therapy, but results in more confident results regard drug safety. Third, the comparison of your results with other second line studies in MPM is difficult because patients' characteristics, employed chemotherapy regimens, methods of response evaluation, and the time from primary diagnosis to start of second line chemotherapy do vary widely.

## Conclusion

Patients with relapsed MPM, refractory to pemetrexed/platin-pretreatment may benefit from a consecutive therapy with oxaliplatin and gemcitabine with a disease control rate of 45% with the absence of WHO grad 4-toxicities. Since now no recommendations based on prospective, randomized trials exist regarding further therapy in these patients, oxaliplatin/gemcitabine can be perceived as an option until these data are available. Since the incidence of MPM will even increase over the next 15 years all centers treating patients with MPM will need to establish also a structured approach for further therapy of patients initially subjected to pemetrexed/platin combination chemotherapy.

## Competing interests

The authors declare that they have no competing interests.

## Authors' contributions

Athanasios Xanthopoulos was responsible for collection of patient data, initial data analysis and contributed to the manuscript. Torsten Bauer contributed to the study design and helped writing the manuscript. Torsten Blum performed final statistical analysis and drafted the manuscript. Jens Kollmeier and Nicolas Schönfeld included patients, helped with data collection and analysis and reviewed the manuscript. Monika Serke guided therapies, supervised data collection, kept the continuously growing database and edited the manuscript.

All authors read and approved the final manuscript.
